# Bovine Brucellosis in Dairy Herds in Mali and Niger, 2024: Apparent Seroprevalence and Epidemiological Factors

**DOI:** 10.3390/pathogens15070764

**Published:** 2026-07-21

**Authors:** Abel S. Biguezoton, Chaka Traore, Haladou Gagara, Der Dabire, Zakaria Bengaly, Mahaman Maaouia Abdou Moussa, Raïssa Bakinahe Ntamukunzi, Kader Issoufou, Maïmouna Ousmane, Claude Saegerman, Marcella Mori

**Affiliations:** 1 Vector-Borne Diseases and Biodiversity Unit (UMaVeB), International Research and Development Centre on Livestock in Sub-Humid Areas (CIRDES), Bobo-Dioulasso BP 454, Burkina Faso; dsdabire@yahoo.fr (D.D.); zakaria.bengaly@cirdes.org (Z.B.); 2 Laboratoire Central Vétérinaire (LCV), Bamako BP 2295, Mali; traorechaka2000@yahoo.fr; 3 Laboratoire Central de l’élevage (LABOCEL), Niamey BP 485, Niger; haladoug@yahoo.fr; 4 Faculté d’Agronomie (FA), Université Abdou Moumouni (UAM), Niamey BP 10896, Niger; maaouia_abdou@yahoo.fr; 5 Sciensano, Belgian Institute for Health, 1050 Brussels, Belgium; raissa.bakinahentamukunzi@sciensano.be; 6 Belgian Agency for International Cooperation (Enabel), Niamey BP 12 987, Niger; kaderiss2006@yahoo.fr (K.I.); maimounaousmane67@gmail.com (M.O.); 7 Research Unit in Epidemiology, Risk Analysis and Biosecurity Applied to Veterinary Sciences (UREAR-ULiege), Fundamental and Applied Research for Animals & Health (FARAH) Center, Faculty of Veterinary Medicine, University of Liege, 4000 Liege, Belgium; claude.saegerman@uliege.be

**Keywords:** bovine brucellosis, dairy herds, Mali, Niger, seroprevalence, epidemiological factors

## Abstract

As part of the PRISMA project, which promotes research and innovation for productive, resilient, and healthy agro-pastoral systems in West Africa, a cross-sectional survey was conducted to assess the apparent seroprevalence and investigate epidemiological factors associated with bovine brucellosis in dairy herds. The survey was carried out in Mali (Bamako, Koulikoro, Mopti, and Sikasso regions) and Niger (Tahoua, Dosso, and Tillabéry regions). A total of 1230 animals from 82 herds were tested, including 645 animals from 43 herds in Mali and 585 animals from 39 herds in Niger. At the herd level, apparent seroprevalence was significantly higher in Mali than in Niger (37.21% vs. 15.38%). Within each country, animal-level seroprevalence varied across region, with the highest levels observed in Bamako (11.11%) and Mopti (7.62%) in Mali, and Dosso (6.67%) in Niger. High within-herd prevalence (>20%) was identified in only one herd per country. The higher overall herd-level seroprevalence in Mali was primarily driven by a greater number of herds with low (≤10%) within-herd seroprevalence. Univariate risk factor analysis at the animal level identified a borderline trend toward higher odds of seropositivity in crossbred cattle than other breeds (OR = 1.95, 95% CI: 1.00–3.81, *p* = 0.0503). In addition, a classification tree analysis highlighted seven exploratory signals of the positive herd status. These included four animal/management-level factors—herd region, environmental disposal of aborted placentas, the number of lactating cows (as a proxy for animal density), and reported herd abortions—as well as three human-level signals within the household: a history of miscarriage, experiencing heavy night sweats, and suffering from undulant fever. In conclusion, this survey provides new insights into bovine brucellosis and its epidemiology in dairy herds in Mali and Niger, confirming established high-prevalence areas such as Bamako and identifying new zones of concern, such as Mopti and Dosso. Our findings underscore that bovine brucellosis remains an important public health and economic threat in both countries. Given that the identified positive regions are hubs for milk production and consumption, these data may support health authorities in implementing targeted surveillance, control, and prevention strategies under a One Health approach.

## 1. Introduction

Brucellosis is a major bacterial zoonotic disease that severely impacts both human health and livestock productivity globally [1,2,3]. Recent conservative estimates indicate a global annual incidence of 1.6–2.1 million new human cases, with the highest burdens reported in Asia and Africa. However, when risk is assessed at the regional level by integrating incidence data with populations at risk, Africa emerges as the continent facing the greatest overall threat, particularly within equatorial zones of East and West Africa [4]. Considerable efforts have been dedicated to advancing the understanding of brucellosis across the continent. A recent systematic review and meta-analysis of brucellosis seroprevalence estimated a pooled livestock and human seroprevalence of 6.20% (95% confidence interval—[CI]: 4.90–7.83) for West Africa [5]. Yet, due to strict study inclusion criteria, this estimate relied on a single human study from Togo and livestock data from a restricted subset of West African countries—specifically Benin (n = 1), Burkina Faso (n = 1), Ghana (n = 2), Guinea (n = 2), Côte d’Ivoire (n = 2), Niger (n = 1), and Togo (n = 1)—while data from Mali were completely lacking. These findings highlight two critical gaps: first, human brucellosis data remain critically scarce in West Africa, despite the disease being recognized as a leading cause of non-malarial febrile illnesses across Africa [6]; second, comprehensive epidemiological data on livestock brucellosis remain fragmentary and are not systematically collected across temporal and geographical scales, including in Mali and Niger [5]. In these two countries, logistical challenges, political instability, and resource constraints further hamper routine surveillance efforts.

In Mali and Niger, agro-pastoral systems are vital to food security and rural livelihoods, particularly driven by the production of bovine milk and dairy derivatives. The cattle sector contributes substantially to national gross domestic products (GDPs) and represents a cornerstone of livestock production within the Economic Community of West African States (ECOWAS/CEDEAO). Beyond their economic significance, cattle constitute one of the primary reservoirs driving human brucellosis. Human infection may occur through direct contact with infected animals, aborted materials, or contaminated fluids; however, the consumption of raw milk and unpasteurized dairy products remains the predominant transmission pathway in endemic areas. This route is highly relevant in Niger, where raw milk consumption is widespread and bovine brucellosis persists as a deeply entrenched issue in rural communities [7]. Furthermore, structural vulnerabilities, such as the limited implementation of farm-level biosecurity measures, continue to exacerbate transmission risks at the human–animal interface [8].

Despite the existence of national epidemiological surveillance networks for priority animal diseases (EPIVET-Mali and RESEPI-Niger) [9], sparse epidemiological data and large-scale structured surveys on bovine brucellosis remain unavailable for both countries. In Mali, bovine brucellosis prevalence is predominantly documented through passive surveillance data or isolated, localized studies. For instance, historical prevalence was estimated at only 0.27% in Moorish Zebu cattle within the Nara Circle [10], contrasting sharply with an apparent prevalence of 22.08% reported in the intensive dairy basins of the Bamako district [11]. In addition, cattle passive surveillance data collected throughout the country between 2007 and 2013 highlighted fluctuating prevalence estimates ranging from a minimum of 5.17% (2009) to a maximum of 19.77% (2013) [12]. In humans, one study conducted on 150 febrile patients at a clinic in the Mopti region between June and August 2007, identified high seropositive rates for brucellosis against agglutination of Wright antigen of *Brucella melitensis* (58%) and *B. abortus* (49%), identifying housewives, traders, butchers and consumption of raw milk as risk factors [13]. Another cross-sectional study in Mopti determined a seroprevalence of 7.43% by BrucellaCAPT and 6.02% by PCR, with *B. abortus* identified as the predominant species in PCR-positive samples [14]. In Niger, brucellosis is known to span all livestock production systems and is endemic in cattle, posing a continuous spillover risk to human populations [7,15,16]. The few existing studies have concentrated heavily on the regions of Niamey, Tillabéri, and Zinder [7], reporting apparent seroprevalence of 2.0% (95% CI: 1.1–2.9%), 1.8% (95% CI: 1.2–2.5%), and 4.6% (95% CI: 3.1–6.2%) across urban, peri-urban and rural production frameworks, respectively. No published data on human brucellosis are currently available for Niger. Consequently, substantial portions of both countries remain epidemiologically undercharacterized, with limited baseline data available.

To address these data gaps, the present cross-sectional survey was conducted under the auspices of the European Union-supported PRISMA project, which aims to foster research and innovation for productive, resilient and healthy agro-pastoral systems in West Africa (specifically targeting Mali, Niger, and Burkina Faso) [17]. This study aimed to estimate the apparent seroprevalence of bovine brucellosis in dairy herds across Mali and Niger and to identify key herd- and animal-level risk factors associated with seropositivity. Given the socioeconomic importance of dairy production and localized milk consumption, this survey deliberately targeted major dairy basins characterized by intensive livestock activity and high human–animal interactions. The resulting data provide a critical, updated epidemiological baseline for Mali and a more comprehensive, multi-regional overview of the disease situation in Niger. Ultimately, these findings are intended to equip national veterinary and public health authorities with the evidence needed to reinforce targeted surveillance, control and prevention strategies against bovine brucellosis under a unified One Health approach.

## 2. Materials and Methods

### 2.1. Cattle Population in Mali and Niger

Mali possesses one of the largest livestock populations in West Africa. Its cattle herd, estimated at over 14 million, represents a major component of the national livestock sector, with the highest cattle densities concentrated in the Mopti region, a key agro-pastoral hub. Similarly, Niger hosts a cattle population exceeding 20 million head, including nearly 4 million milking cows, which are predominantly managed within traditional agro-pastoral systems.

### 2.2. Study Area and Sample Size Calculation

**Figure 1:**
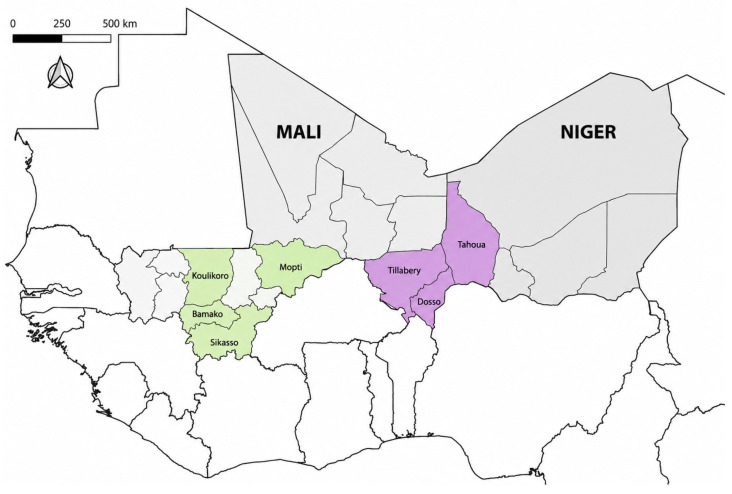
Mapping of the study area of this survey. The highlighted regions correspond to the dairy production basins selected for sampling. In Mali, the surveyed regions included Bamako, Koulikoro, Mopti, and Sikasso, while in Niger the surveyed regions included Tahoua, Dosso, and Tillabéry.

The data analyzed in this survey were generated as part of a comprehensive sero-epidemiological survey conducted between June and September 2024 under the auspices of the PRISMA project. The research targeted dairy cattle farms across four regions in Mali (Bamako, Koulikoro, Mopti, and Sikasso) spanning 43 locations, and three regions in Niger (Tahoua, Dosso, and Tillabéry) covering 39 locations. These regions were selected based on local milk consumption patterns and their status as primary dairy production basins within each country (Figure 1).

The survey followed a multistage probabilistic sampling design adapted to local operational constraints. Within each region, sampling locations (villages) were selected to maximize spatial coverage while accounting for field accessibility and security considerations. Within each selected location, one herd was randomly chosen, and 15 animals were randomly sampled from that herd. Because only one herd was sampled per village, each location corresponds directly to an individual herd. The epidemiological survey unit (herd) was defined as a group of sedentary, lactating cows managed collectively, owned by a single household, and maintained at the same geographic site. The target sample size was determined based on the estimated population of lactating females in each country and region (Table 1), an expected brucellosis seroprevalence of 10% derived from baseline literature [7], an allowable error margin of 1.75%, and a 95% CI. The overarching study design was developed by a multidisciplinary team of epidemiologists and formally approved during methodological workshops in 2022. These workshops involved leading One Health stakeholders representing the human public health, veterinary medicine, epidemiology, and diagnostic laboratory sectors of both nations.

### 2.3. Rose Bengal Testing (RBT)

Whole-blood samples were collected in the field using serum separator tubes, allowed to clot at ambient temperature, and transported under cold chain conditions to national laboratories. Upon arrival, tubes were centrifuged at 3000 rpm, and the supernatant serum was aliquoted and stored at −20 °C until diagnostic processing. Serum samples were evaluated using the Pourquier Rose Bengale Ag kit (IDEXX Laboratories, Saint-Denis, France), which utilizes a standardized suspension of inactivated *B. abortus* (strain Weybridge 99), following the manufacturer’s protocols and World Organization for Animal Health (WOAH) guidelines [18]. Briefly, 50 µL of antigen and 50 µL of test serum were dispensed side by side to the same well of a 24-well round-bottom glass plate. A specialized multi-pronged plastic comb was used to mix the antigen and serum simultaneously across wells. The plate was rocked continuously to observe agglutination over a strict 4 min incubation window, after which positive or negative reactions were immediately recorded. All diagnostic runs were performed alongside concurrent reference positive (serum from *B. abortus*-experimentally infected cattle [19]) and negative controls (fetal bovine serum; GE Healthcare, Machelen, Belgium). Reagent batches were certified by the European Reference laboratory for Brucellosis [20], procured via Belgium, and distributed directly to the respective local diagnostic facilities. Under field conditions, the reported diagnostic sensitivity of the RBT ranges from 80% to 90%, with a specificity between 95% and 98% in cattle [21].

### 2.4. Determination of Apparent Seroprevalence

Apparent seroprevalence at both the animal and herd levels was calculated as the proportion of positive test results relative to the total number of samples evaluated. Associated 95% CIs were calculated using the Wilson score method [22]. An individual animal was classified as seropositive if visible macroscopic agglutination was observed during the RBT. A herd was defined as seropositive if at least one animal within that herd tested positive.

### 2.5. Epidemiological Questionnaire Survey

A standardized epidemiological questionnaire was administered to collect information on animal characteristics, herd management practices, and household-level human behaviors and symptoms in order to identify potential risk factors associated with disease transmission (the complete list of variables is provided in Table A1). Data were collected through face-to-face interviews conducted in the local language by trained veterinarians and recorded electronically in real time using handheld tablets.

### 2.6. Statistical Analysis and Spatial Mapping

Statistical analyses were performed using Epitool [22], R version 4.2.3 [23] and STATA SE version 14.2 (StataCorp, College Station, TX, USA). Apparent animal- and herd-level seroprevalences, alongside within-herd seroprevalence, were compared across countries and regions using Pearson’s Chi-squared tests. Cohen’s Kappa coefficient was used to quantify inter-rater agreement for categorical parameter while adjusting for chance agreement [24]. Confidence intervals were calculated according to the Wilson efficient-score method, corrected for continuity. Kappa values were interpreted according to standard benchmarks: <0.2, slight agreement; 0.2–0.4, fair agreement; 0.4–0.6, moderate agreement; 0.6–0.8, substantial agreement; and >0.8, almost perfect agreement.

Univariate analyses exploring associations between potential risk factors and brucellosis seropositivity were performed at multiple spatial levels (combined countries, individual country level, region level, and pooled selected regions) for both animal and herd data. The serological outcome variable was treated as a binary variable (positive vs. negative). Animal-level models evaluated four categorical variables: sex, age group, breed, and species. Herd-level models assessed eighteen variables (Table A1), of which sixteen were binary. The household sanitary conditions variable was stratified into three tiers (1 = Good; 2 = Moderate; 3 = Poor), and the total number of lactating females within the herd was categorized into four intervals (1 = <10; 2 = 10–19; 3 = 20–29; 4 = 30–39).

Variables demonstrating marginal or strong associations in the univariate screening (*p* ≤ 0.10) were advanced into multivariable logistic regression models. At the individual animal level, the multivariable model adjusted for breed category (crossbred vs. other breeds) and country of origin. At the herd level, the multivariable framework incorporated placenta disposal practices and the presence of animal assembly areas. Adjusted odds ratios (aOR) were calculated to quantify risks while controlling for confounding effects. Statistical significance was defined at *p* < 0.05, whereas *p*-values between 0.05 and 0.10 were considered indicative of a statistical trend.

Classification tree analysis (CTA) was subsequently implemented to resolve direct and indirect explanatory pathways influencing herd brucellosis status. Classification and regression tree (CART) modeling is a non-parametric machine learning technique based on recursive binary partitioning. This approach systematically splits a population into increasingly homogeneous subgroups relative to the target dependent variable (for architectural details, see [25]). The CART analysis carries out cross-validation, growing optimum trees on data sub-groups, then calculating the error rates based on the parts of the complete dataset not used. The CART analysis divides all the data into ten almost equal parts selected at random, with each part containing a similar distribution of data from the populations under consideration (namely notification or non-notification of abortions by the farmer). The analysis then uses the first nine datasets (9/10) to construct the largest possible tree and uses the last part of the data (1/10) to estimate the error rate of the selected tree. The process is repeated using different combinations of the nine remaining data subsets and a different data subset to test the resulting tree. This process is repeated until each 1/10 data subset has been used to test a tree developed using the remaining nine dataset subsets (9/10). The results of the ten mini-tests are then combined to calculate the error rates for each possible size of tree. These error rates are then applied to prune the tree that was developed using all the data. The outcome of this complex process is a set of relatively reliable independent estimates of the accuracy of the clinical decision tree prediction. For each node in a tree generated by CART, the main separator is the variable that best divides the node, thereby maximizing the purity of the resulting nodes. To test the diagnostic power of the final decision tree generated, a receiver operating characteristic (ROC) was employed both for the original dataset used to construct the tree (training data) and for the dataset used to test the performance of the decision tree (test data) [25].

Spatial maps were generated using QGIS software (version 3.44, [26]) using publicly available boundaries [27].

### 2.7. Ethical Requirements

The study protocols and field instruments were formally vetted and approved by respective institutional review board: the Ethics Committee of the University of Sciences, Techniques and Technologies of Bamako (USTTB) in Mali and the National Ethics Committee for Health Research (CNERS) of Niger. Following institutional reviews, protocols were refined, and final written ethical clearances were issued in August and June 2023 under reference numbers 2023/190/CE/USTTB and 35/2023/CNERS, respectively.

## 3. Results

### 3.1. RBT Training and Test Performance Validation

Prior to launching the survey, the diagnostic proficiency for RBT at the two participating national laboratories (LCV-Mali and LABOCELL-Niger) was evaluated via an interlaboratory comparison using a standardized panel of blinded reference sera. The initial baseline assessment revealed discrepancies in diagnostic performance between laboratories. While the first laboratory demonstrated perfect concordance with the reference standard (κ = 1.00), the second laboratory exhibited significantly lower diagnostic agreement (κ = 0.444). To address these technical shortcomings, two capacity-building training interventions were implemented: an intensive on-site workshop in Niamey (April 2023) and a follow-up interactive online session (June 2023). These sessions focused on harmonizing procedural methodologies, standardizing diagnostic reading criteria using certified reference standards, and validating technical performance through joint evaluation by analysts from the Belgian Brucellosis Reference Centre and local laboratory staff. Following these interventions, a repeat proficiency panel confirmed that the second laboratory achieved perfect diagnostic alignment with the expected reference outcomes (κ = 1.0; Table A2).

Subsequently, a method validation comparison between the standardized RBT and an indirect enzyme-linked immunosorbent assay (ELISA) was performed on a representative serum subset (n = 407) (Table A3). Given the almost perfect agreement observed between the two diagnostic modalities (κ = 0.883; *p* < 0.05), coupled with the superior cost-effectiveness and field-level operational feasibility of the agglutination platform in low- and middle-income settings, RBT was selected as the sole diagnostic framework for full dataset analysis.

### 3.2. Apparent Animal- and Herd-Level Seroprevalence and Mapping of Bovine Brucellosis in Mali and Niger

A total of 1230 animals from 82 herds were tested across Mali and Niger. Overall, apparent animal-level seroprevalence was higher in Mali than in Niger; however, this cross-border divergence achieved statistical significance only when aggregated at the herd level (37.21% vs. 15.38%, *p* = 0.048) (Table A4).

**Figure 2:**
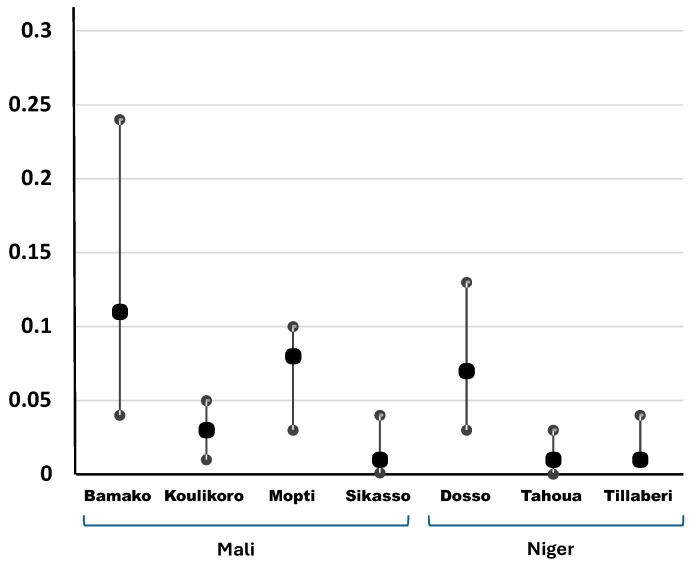
Apparent animal prevalence with 95% confidence interval of brucellosis in different regions of Mali and Niger.

Substantial intra-country variations in apparent seroprevalence were observed across administrative zones. In Mali, where 645 animals from 43 herds were evaluated, apparent animal-level seroprevalence varied significantly by region (*p* = 0.0017), whereas herd-level variations did not reach statistical significance. The highest animal-level seroprevalence was clustered in Bamako district (11.11%; 5/45; 95% CI: 4.84–23.5%), followed by Mopti (7.62%; 8/105; 95% CI: 3.91–14.32%), Koulikoro (2.73%; 9/330; 95% CI: 1.44–5.10%), and Sikasso (1.21%; 2/165; 95% CI: 0.33–4.31%) (Table A4 and Figure 2).

Similarly, in Niger, where 585 animals across 39 herds were tested, apparent animal-level seroprevalence differed significantly by region (*p* = 0.0008) but remained statistically uniform at the herd level. The highest animal seroprevalence was detected in the Dosso region (6.67%; 8/120; 95% CI: 4.00–13.64%), followed by Tillabéry (1.48%; 4/270; 95% CI: 0.58–3.75%) and Tahoua (0.51%; 1/195; 95% CI: 0.09–2.85%) (Table A4 and Figure 2).

**Figure 3:**
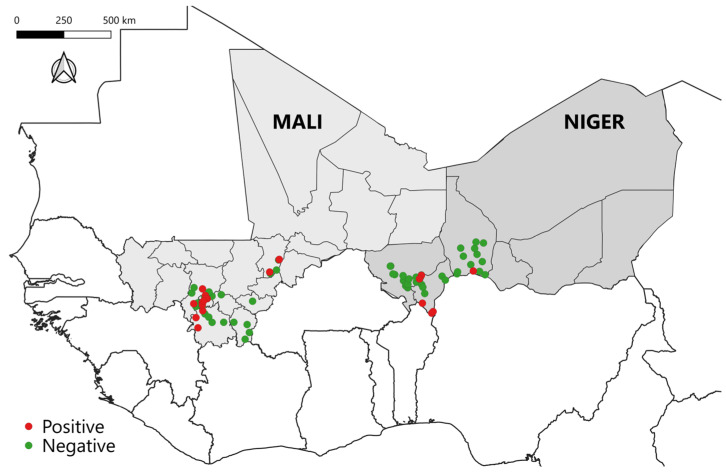
The geographic distribution of herds included in the cross-sectional survey in Mali and Niger. Green and red dots represent seronegative and seropositive herds, respectively.

The geographical distribution of positive and negative herds highlights regional differences in herd infection status (Figure 3).

Stratification of within-herd seroprevalence profiles highlighted distinct transmission dynamics between the two study populations (Table 2). Among the seropositive cohorts in Mali, eleven herds exhibited low (≤10%) within-herd seroprevalence, four showed moderate seroprevalence, and a single herd displayed high within-herd seroprevalence (>20%), with the highest within-herd focus localized in Horokinde (Mopti region). In Niger, two herds displayed low within-herd seroprevalence, three were classified as moderate, and one herd exhibited a high seroprevalence profile, with the latter pinpointed in Gaya (Dosso region).

Cumulatively, Bamako and Mopti in Mali, alongside Dosso in Niger, presented the highest apparent seroprevalence values, identifying them as hotspots of increased *Brucella* circulation within the local dairy value chain. The elevated overall herd-level seroprevalence recorded in Mali relative to Niger was statistically driven by a significantly higher proportion of herds with low within-herd seroprevalence (*p* < 0.05).

### 3.3. Exploratory Epidemiological Signals of Herd Brucellosis

Risk factor analyses were performed using pooled data from both countries at the animal level, whereas herd-level analyses were conducted at different spatial levels (pooled datasets, country specific, and isolated regional levels) (Table A5).

Univariate logistic regression models identified a borderline trend toward higher odds of seropositivity in crossbred cattle compared to local indigenous breeds (OR = 1.95, 95% CI: 1.00–3.81, *p* = 0.0503). The same association was observed for the category *Bos taurus indicus × Bos taurus taurus*, which corresponds to the same group of crossbred animals and therefore should not be interpreted as independent findings. However, this indicator requires cautious interpretation due to its marginal significance and wide confidence interval. Age classifications and sex showed no statistical association with seropositivity. At the herd level, traditional management practices—specifically open-air carcass dumping and poor placenta disposal—demonstrated weak, non-significant positive associations with herd seropositivity.

In the final multivariable logistic regression analysis, which adjusted for all baseline parameters displaying univariable significance or trends (*p* ≤ 0.10), crossbred cattle retained an independent trend toward higher odds of animal-level seropositivity (aOR = 1.73). At the herd level, the environmental disposal of placentas remained tied to higher odds of herd-level positivity (aOR = 2.55), whereas the utilization of dedicated animal assembly areas correlated with lower odds (aOR = 0.32). However, upon multivariable adjustment, none of these specific indices maintained independent statistical significance (*p* > 0.05).

**Figure 4:**
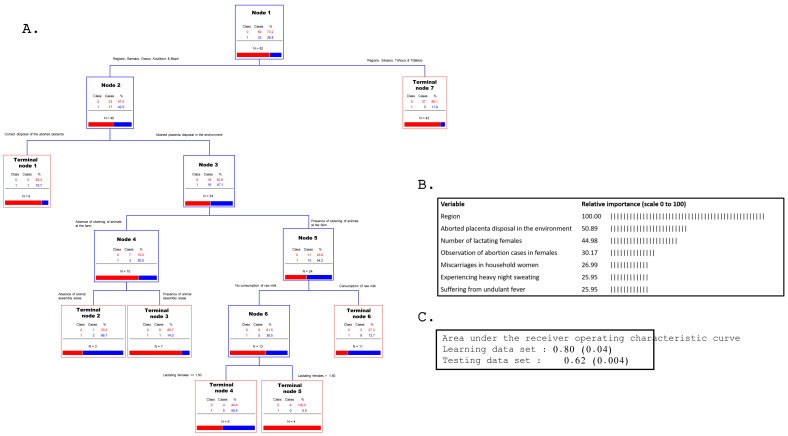
Classification tree analysis (CTA) of predictive factors linked to binary herd-level brucellosis status. (**A**) Classification tree; (**B**) relative importance rankings of selected exploratory signals; (**C**) receiver operating characteristic (ROC) validation curves displaying area under the curve (AUC) values alongside their corresponding standard error [e.g., AUC (SE)] for both the internal training and evaluation test datasets. Class 0 indicates seronegative herds status (red), and Class 1 represents confirmed seropositive herds status (blue). Node metrics display raw herd counts and total group percentages.

To uncover non-linear, hierarchical relationships influencing herd-level status, a non-parametric CTA was deployed (Figure 4). The primary operational split identified by the CTA was the administrative region (relative importance [RI] = 100; considering a scale from 0 to 100). This was followed hierarchically by the environmental disposal of aborted placentas (RI = 50.89), the total number of lactating cows (RI = 44.98) and a documented history of clinical abortions in female animals (RI = 30.17).

The tree structure also selected three human compartment variables within the livestock-owning households as parallel exploratory signals: a history of spontaneous miscarriages in household women (RI = 26.99), reported patterns of heavy night sweats (RI = 25.95), and clinical episodes of undulant fever (RI = 25.95). The final predictive tree architecture achieved an area under the receiver operating characteristic curve (AUC-ROC) of 0.80 for the internal training data array and an AUC-ROC of 0.62 for the cross-validation testing dataset.

## 4. Discussion

This survey provides an updated overview of the epidemiological situation of bovine brucellosis in selected high-consumption, major dairy production basins of Mali and Niger. Our findings confirm the endemic presence of bovine brucellosis in both countries and reveal notable spatial variation in animal prevalence at the regional level, pointing to heterogeneous transmission dynamics.

### 4.1. Apparent Prevalence at Herd and Animal Level, and Within-Herd Prevalence

In Mali, the highest apparent animal seroprevalence was detected in the Bamako region, reaching 11.11%. This finding aligns with the historically high levels of seropositivity reported in the area. Notably, data from routine analyses conducted at LCV between 1989 and 1990 using indirect ELISA (iELISA) reported an individual seropositivity rate of 19.7% in cattle from the Bamako district [28] and 23.3% in 1994 [12]. The high animal-level prevalence observed in our survey correlated with a substantial herd-level prevalence of 66.67%. Bamako—alongside Lomé (Togo), Bujumbura (Burundi), and Bamenda (Cameroon)—has previously been identified among the zones with the highest herd seroprevalence levels in dairy cattle milk across West and Central Africa, marking it as an area of elevated risk for human brucellosis [16]. Furthermore, microbiological quality assessments of cattle milk in Bamako using iELISA and milk ring test have documented brucellosis seroprevalence levels up to 30% [29], reinforcing the hypothesis of sustained pathogen circulation in this region.

Although the exact drivers of this high seroprevalence were not explicitly identified in this survey, several structural factors likely contribute to transmission. Bamako serves as a primary livestock hub at both national and transboundary levels, particularly for trade with neighboring coastal countries. The influx of cattle, predominantly from the Ségou and Mopti regions, routinely exceeds the outflow from Bamako—especially during the rainy season and major religious festivals [30]. This intensified animal movement may increase opportunities for herd mixing and contacts between animals of unknown health status [31]. Furthermore, the proliferation of specialized, semi-intensive livestock production systems in and around expanding urban centers creates conditions favorable to pathogen persistence, primarily through closer and more frequent animal contacts [32].

The second region of high seroprevalence in Mali was Mopti, where apparent animal seroprevalence reached 7.62%. These values are consistent with animal and herd prevalence data from 1991 [28], despite methodological differences between the two studies. In contrast, the 1991 survey reported much higher animal seroprevalence levels in the Koulikoro and Sikasso regions (43% and 26%, respectively) which were not replicated in our findings. These discrepancies may stem from temporal shifts in disease circulation or reflect broader changes in livestock distribution. Over recent decades, a gradual relocation of livestock from central regions toward southern zones and neighboring countries like Côte d’Ivoire is documented [33]. This shift, driven by climate change, mounting pressure on grazing lands, and dwindling natural resources, may explain the lower prevalence captured in our study for these areas.

In Niger, the highest apparent animal seroprevalence was observed in the Dosso region, though it remained relatively low at 6.67%. Recent cross-sectional estimates of bovine brucellosis in Niger, obtained via iELISA in the urban community of Niamey, its surroundings, and the communities of Balleyara and Torodi, reported apparent animal seroprevalence ranging from 1.8% to 4.6% across urban, peri-urban, and rural settings [7]. These findings are consistent with the modest seroprevalence observed in our study, though our estimates for Dosso were slightly higher.

Historical surveillance in Niger highlights significant temporal fluctuations in disease data. For instance, average seroprevalence levels reached up to 30% in 1980–1981 [34], whereas country-wide passive surveillance effort in 1989 using the RBT on approximately 3000 serum samples yielded a drastically lower prevalence of around 1.4% [35]. These stark variations between studies likely reflect temporal fluctuations in pathogen circulation alongside differences in sampling designs, diagnostic methodologies, and target populations. The comparatively elevated seroprevalence in Dosso may be attributed to its strategic position as a commercial crossroads. Dosso intersects major trade routes linking Nigeria and Niger—particularly for the importation of specific cattle breeds—as well as corridors connecting Niger to southern nations such as Côte d’Ivoire and Burkina Faso [36]. Such intensive cross-border movements may facilitate contact between disparate herds of unknown health status, prompting the introduction and propagation of the disease.

Within-herd prevalence was generally low across most herds in both nations, with only one herd per country exceeding 20% prevalence. Discrepancies between the two countries were primarily driven by the distribution of herds with low-level within-herd seropositivity. Similar epidemiological patterns have been documented in other African contexts, where brucellosis frequently persists at low within-herd prevalence in commercial and agro-pastoral framework compared to more extensive pastoral systems [37]. The higher frequency of low within-herd seroprevalence observed in Mali as compared to Niger points to a pattern of persistent, endemic infection characterized by low-level *Brucella* circulation that avoids generating widespread, acute outbreaks [38,39,40]. Conversely, in settings like Mali and Niger, where livestock vaccination programs are absent, low-level prevalence can also signal the early stages of pathogen introduction into naïve herds [40]. Regardless of the underlying mechanism, even a small cohort of infected animals can sustain the pathogen within a population through shedding during reproductive events. This may contribute to the persistence of infection and represent a continuing public health concern, particularly in traditional dairy chains where raw milk consumption remains a tradition.

### 4.2. Epidemiological Factors and Exploratory Signals of Bovine Brucellosis

Univariate analysis identified few potential risk or protective factors. At the animal level, crossbred cattle exhibited a borderline trend toward higher odds of seropositivity compared to indigenous breeds (OR = 1.95, *p* = 0.0503). However, this finding must be interpreted with caution due to its wide confidence interval and the borderline statistical significance. Nevertheless, this trend mirrors prior literature indicating that breed type can influence brucellosis, with crossbred cattle often showing higher seroprevalence than native breeds due to variances in husbandry and management [41]. Rather than reflecting true genetic susceptibility, this increased risk is likely management-driven, as crossbred animals are predominantly utilized in semi-intensive or peri-urban dairy systems characterized by higher stocking densities. Our survey did not detect significant associations between seropositivity and age or sex, despite these being well-established risk factors in the broader literature [42,43,44,45]. Generally, female and older animals display higher seroprevalence due to prolonged exposure windows and the cumulative probability of infection over time.

At the herd level, practices such as disposing of placenta and carcasses in open areas were documented but did not emerge as statistically significant risk factors. While inadequate disposal of reproductive waste has been widely flagged as a primary driver of transmission in East and West Africa due to environmental contamination [46], its statistical impact was not confirmed in this specific dataset.

Conversely, the classification tree analysis highlighted region as the primary exploratory signal of herd brucellosis status, underscoring the role of geographical heterogeneity, particularly in Mali. In high-prevalence regions, the disposal of aborted placentas in the open environment acted as a possible discriminator. This aligns with biological expectations, as aborted materials may contain high concentrations of *Brucella* and serve as major sources of environmental contamination and herd reinfection.

The next two exploratory signals of herd status were the number of lactating females and the occurrence of herd abortions. A larger cohort of lactating females typically characterizes intensive dairy operations, where increased animal density and animal-to-animal contact may facilitate transmission. Similarly, the history of abortions is a highly plausible clinical indicator, given that abortion is the hallmark clinical manifestation of bovine brucellosis. The reduction in AUC from the training to the testing dataset points to some degree of overfitting. Therefore, this classification tree analysis should primarily be considered an exploratory tool for highlighting epidemiological signals. Finally, the model selected human-related variables, including histories of miscarriages among household women and clinical signs compatible with human brucellosis. These findings should be regarded as exploratory signals, as dedicated public health and serological investigations were not conducted among household members in this study. However, they support the close epidemiological links between livestock and farming households, reinforcing the necessity of tackling brucellosis through a unified One Health framework.

### 4.3. Limitation of the Study

Some limitations should be considered when interpreting this study’s results:

- Target population bias: The survey was restricted exclusively to sedentary lactating cows. Consequently, the data do not fully mirror the broader cattle sector, as non-lactating cows, bulls, and young stock were excluded, which may have skewed overall herd-level prevalence estimates. However, this narrow focus was intentional, as the dairy sector represents the primary pathway for zoonotic transmission via the consumption of raw milk and unpasteurized dairy products common in Sub-Saharan Africa [47,48].
- Selection bias: The fixed sampling quota per herd, alongside logistically challenging field conditions, accessibility issues, and security constraints, may have introduced selection bias during herd recruitment, potentially leaving certain remote areas or herds underrepresented.
- Diagnostic constraints: Diagnosis relied on the Rose Bengal Test without systematic confirmatory testing. However, the RBT remains an exceptionally cost-effective, highly field-applicable tool in low- and middle-income countries. It has demonstrated robust diagnostic sensitivity and specificity in African cattle sera [49,50], making it a suitable testing option for resource constrained laboratory settings. To minimize diagnostic error, extensive training and harmonization protocols were executed prior to fieldwork, and parallel ELISA testing was conducted on a subset of samples. Given the nearly perfect agreement observed between the two methods, RBT was deemed reliable for the full analysis. Furthermore, because brucellosis vaccination is not practiced in either Mali or Niger, false positives stemming from vaccine-induced cross-reactivity can be confidently ruled out.
- Study Design: The cross-sectional nature of this survey precludes the establishment of temporal relationships or definitive causal associations between exposure variables and seropositivity. Identified risk factors must therefore be viewed strictly as statistical associations rather than causal determinants. This is particularly true for the borderline association between crossbred animals and seropositivity (OR = 1.95, *p* = 0.0503), where the wide confidence interval and threshold *p*-value signify a degree of statistical instability.

## 5. Conclusions

This survey provides critical, updated data on the epidemiological landscape of bovine brucellosis within the dairy sector of Mali and Niger. Collectively, these findings advance our understanding of disease distribution and offer a baseline to support national authorities and stakeholders in refining surveillance, control, and prevention frameworks. The sustained circulation of bovine brucellosis in major dairy basins highlights the urgent need to integrate animal and human surveillance under a unified One Health strategy particularly where raw milk production and consumption are culturally and economically vital.

To build upon these insights, future research should employ larger sample sizes, integrate concurrent household sampling, and prioritize the collection of viable specimens for the isolation and molecular characterization of circulating *Brucella* strains. Identifying the specific species and genotypes active at the animal–human interface will contribute to understanding the transmission dynamics and pave the way for targeted, cross-sectoral intervention strategies. Ultimately, controlling brucellosis in these regions requires moving beyond isolated livestock interventions toward a coordinated One Health approach that simultaneously protects animal health, secures rural livelihoods, and mitigates zoonotic risk to human populations.

## Figures and Tables

**Table 1:** The estimated number of cattle and lactating cows in the survey region of Mali and Niger.

Country	Region	Number of Cattle	Number of Lactating Cows
Mali	Koulikoro	1,617,000	291,192
	Sikasso	1,796,300	323,480
	Mopti	3,155,500	568,247
	Bamako	37,400	6735
	Sub-total	6,606,200	1,189,654
Niger	Dosso	1,984,437	264,921
	Tahoua	3,838,052	512,378
	Tillabéry	3,526,844	560,768
	Sub-total	9,349,333	1,338,067
	Total	15,955,533	2,527,721

**Table 2:** Within-herd seroprevalence of bovine brucellosis stratified by country and administrative region. Within-herd seroprevalence are categorized into four distinct tiers: null (0%), low (>0–10%), moderate (>10–20%), and high (>20%).

		0%	>0–10%	>10–20%	>20%
Mali	Bamako	1	0	2	0
	Koulikoro	14	7	1	0
	Mopti	3	2	1	1
	Sikasso	9	2	0	0
	Subtotal	27	11	4	1
Niger	Dosso	5	1	1	1
	Tahoua	12	1	0	0
	Tillabéry	16	0	2	0
	Subtotal	33	2	3	1

## Data Availability

The raw data supporting the conclusions of this article will be made available by the authors on request.

## Abbreviations

The following abbreviations are used in this manuscript:

CART	Classification and Regression Tree
CNERS	National Ethics Committee for Health Research
CTA	Classification Tree Analysis
ECOWAS/CEDEAO	West African Economic Community
ELISA	Enzyme-Linked ImmunoSorbent Assay
EU	European Union
GDP	Gross Domestic Product
LABOCELL	Central Livestock Laboratory
LCV	Central Veterinary Laboratory
LVK	Kayes Veterinary Laboratory
PRISMA	Research and Innovation for Productive, Resilient and Healthy Agro-Pastoral Systems in West Africa
QGIS	Quantum Geographic Information System
RBT	Rose Bengal Test
RESEPI	Epidemiological Surveillance Network for Priority Animal Diseases
USTTB	University of Sciences, Techniques and Technologies of Bamako
WOAH	World Organisation for Animal Health

## Appendix A

### Appendix A.1

**Table A1:** List of documented research data collected during the epidemiological survey. Animal, herd, and human-level variables collected through the questionnaire, with their definitions and possible responses.

Type	Level	Variable	Possible Responses
Animal	Animal	Sex	Male; Female
Animal	Animal	Age group	1 = 15–25; 2 = 26–36; 3 = 37–47; 4 = 48–58; 5 = 59–70; 6 = 71–81; 7 = 82–92
Animal	Animal	Breed	Crossbred (Zebu × Taurine), Local breeds (Maure Zebu, Peuhl Zebu or N’Dama), Zebu, Goudali, Azawak, Djelli, Holstein, Mbororo, Kouri, White Fulani.
Animal	Animal	Species	Bos taurus indicus, Bos taurus taurus, Bos taurus taurus X Bos taurus indicus
Herd management	Herd	Presence of animal assembly areas	Yes; No
Herd management	Herd	Household sanitary conditions	1 = Good; 2 = Moderate; 3 = Poor
Herd management	Herd	Number of lactating females present in the herd	1 = <10; 2 = 10–19; 3 = 20–29; 4 = 30–39
Herd management	Herd	Washing hand before milking	Yes; No
Herd management	Herd	Washing of equipment before milking	Yes; No
Herd management	Herd	Washing hand after milking	Yes; No
Herd management	Herd	Washing of equipment after milking	Yes; No
Herd management	Herd	Disposal of carcasses	1 = In the open environment; 0 = Other
Herd management	Herd	Disposal of aborted fetuses	1 = In the open environment; 0 = Other
Herd management	Herd	Disposal of placenta in case of abortion	1 = In the open environment; 0 = Other
Herd management	Herd	Slaughtering of animals at the farm	Yes; No
Animal	Herd	Observation of abortion cases in females of the herd	Yes; No
Animal	Herd	Observation of testicular problems in males (e.g., pain, swelling)?	Yes; No
Animal	Herd	Observation of animals with swelling of the joints (hygromas)?	Yes; No
Human factor	Herd	Milk consumption	1 = Fresh unboiled milk; 0 = Other
Human factor	Herd	Observation of miscarriages in women of the family surroundings?	Yes; No
Human factor	Herd	Suffering from chronic fever (prolonged or undulating) that does not respond to treatment	Yes; No
Human factor	Herd	Experiencing heavy night sweating	Yes; No

### Appendix A.2

**Table A2:** Improvement in the reliability of laboratory procedures and RBT interpretation following personnel training prior to data collection.

Laboratory	Training Stage	Observations	Qualitative Agreement (κ) (95% CI)	*p*-Value
Laboratory 1	Before training	10	1.00 (0.66–1.00)	*p* < 0.01
Laboratory 2	Before training	10	0.444 (−0.07–0.96)	n.s.
Laboratory 2	After training	63	1.00 (0.93–1.00)	*p* < 0.0001

### Appendix A.3

**Table A3:** Comparison between RBT and ELISA results on a subset of sera.

	RBT Positive	RBT Negative	Total
ELISA positive	16	0	16
ELISA negative	4	387	391
Total	20	387	407

### Appendix A.4

**Table A4:** Apparent seroprevalence of brucellosis at the animal and herd level in Mali and Niger. Animal- and herd-level apparent seroprevalence are stratified by country and region, and expressed as percentile with corresponding 95% confidence intervals. *p*-values < 0.05 indicate statistically significant differences between countries and regions.

Country	Region	Animal Tested	Animal Seroprevalence % (n) (95% CI)	*p*-Value	Herds Tested	Herd Seroprevalence % (n) (95% CI)	*p*-Value
Mali	Bamako	45	11.11 (5) (4.84–23.5)	0.0017	3	66.67 (2) (20.77–93.85)	0.2597
	Koulikoro	330	2.73 (9) (1.44–5.10)		22	36.36 (8) (19.73–57.05)	
	Mopti	105	7.62 (8) (3.91–14.32)		7	57.14 (4) (25.05–84.18)	
	Sikasso	165	1.21 (2) (0.33–4.31)		11	18.18 (2) (5.14–47.7)	
Subtotal		645	3.72 (24) (2.51–5.48)		43	37.21 (16) (24.38–52.14)	
Niger	Dosso	120	6.67 (8) (4.00–13.64)	0.0008	8	37.50 (3) (13.68–69.43)	0.1459
	Tahoua	195	0.51 (1) (0.09–2.85)		13	7.69 (1) (1.37–33.31)	
	Tillabéry	270	1.48 (4) (0.58–3.75)		18	11.11 (2) (3.1–32.8)	
Subtotal		585	2.22 (13) (1.3–3.76)		39	15.38 (6) (7.25–29.73)	
Mali vs. Niger		1230		0.1708	82		0.0479

### Appendix A.5

**Table A5:** Risk factors for bovine brucellosis at the animal and herd levels in Mali and Niger: results of univariate analyses. Animal-level results are based on individual animals, while herd-level results are based on herd management practices. Underlined *p*-values indicate a statistical trend (0.05 ≤ *p* < 0.10). OR = Odds Ratio, CI = Confidence Interval (95%), y-years.

Level	Variable Description	Category	Country/Region	Univariate Logistic Regression		
				OR	95% CI	*p*-Value
Animal	Breed	Djelli	Mali + Niger	0.85	0.35–2.05	0.7126
Animal	Breed	Local	Mali + Niger	1.05	0.52–2.11	0.8975
Animal	Breed	MBororo	Mali + Niger	1.23	0.43–3.54	0.7007
Animal	Breed	Crossbred (metisse)	Mali + Niger	1.95	1–3.81	0.0503
Animal	Species	*Bos taurus indicus*	Mali + Niger	0.5	0.24–1.04	0.0653
Animal	Species	*B. taurus indicus* × *B. taurus taurus*	Mali + Niger	1.95	1–3.81	0.0503
Animal	Species	Mixte	Mali + Niger	1.05	0.52–2.11	0.8975
Animal	Age	1–5 y	Mali + Niger	0.77	0.4–1.48	0.4346
Animal	Age	6–10 y	Mali + Niger	1.39	0.72–2.68	0.3216
Animal	Age	11–15 y	Mali + Niger	0.66	0.09–4.93	0.6879
Animal	Sex	Female	Mali + Niger	1.52	0.71–3.25	0.2831
Animal	Sex	Male	Mali + Niger	0.66	0.31–1.41	0.2831
Herd	Presence of animal assembly areas	Yes vs. No	Mali + Niger	0.24	0.05–1.16	0.075
Herd	Placenta disposal	Litter vs. Other	Mali + Niger	3.0	0.90–9.96	0.073
Herd	Cadaver disposal	Litter vs. Other	Mali + Niger	2.3	0.60–8.84	0.224
Herd	Raw milk consumption	Raw vs. Other	Mali + Niger	1.14	0.38–3.39	0.81
Herd	Presence of animal assembly areas	Yes vs. No	Mali	0.24	0.04–1.50	0.127
Herd	Cadaver disposal	Litter vs. Other	Mali	0.58	0.03–9.91	0.705
Herd	Raw milk consumption	Raw vs. Other	Mali	2.42	0.68–8.64	0.172
Herd	Placenta disposal	Litter vs. Other	Niger	1.15	0.18–7.33	0.882
Herd	Cadaver disposal	Litter vs. Other	Niger	1.67	0.27–10.39	0.584
Herd	Placenta disposal	Litter vs. Other	Dosso	8.00	0.31–206.38	0.210
Herd	Cadaver disposal	Litter vs. Other	Dosso	3.00	0.15–59.89	0.472
Herd	Placenta disposal	Litter vs. Other	Bamako + Mopti + Dosso	6.4	0.55–74.89	0.139
Herd	Cadaver disposal	Litter vs. Other	Bamako + Mopti + Dosso	4.0	0.33–48.66	0.277
Herd	Raw milk consumption	Raw vs. Other	Bamako + Mopti + Dosso	1.0	0.14–7.10	1.0
